# Parenting stress and risk of child maltreatment during the COVID-19 pandemic: A family stress theory-informed perspective

**DOI:** 10.1177/2516103220967937

**Published:** 2020-10-20

**Authors:** Qi Wu, Yanfeng Xu

**Affiliations:** 1 115456 Arizona State University, USA; 2 143869 University of South Carolina, USA

**Keywords:** COVID-19, family stress theory, heightened child maltreatment risk, parental resources, parenting stress, perception and coping

## Abstract

The risk of child maltreatment is heightened during the pandemic due to multiple COVID-19 related stressors, such as physical and mental health concerns, economic stress, challenges in homeschooling, marital conflicts and intimate personal violence, and intensified child–parent relationships. Both parental internal (e.g., parenting styles) and external resources (e.g., social support), and parental perceptions toward stressors will affect how parents cope with these stressors, which may exacerbate or mitigate the risk of child maltreatment. Guided by family stress theory, this article identifies COVID-19 related stressors at the family level, and further elaborates on how these stressors are associated with child maltreatment via parents’ resources, perceptions, and coping strategies. Implications for future practice and research are discussed.

## Introduction

The novel coronavirus disease, 2019 (COVID-19), has spread rapidly worldwide, and the fallout from the pandemic is still unfolding (Yip & Chau, 2020). Policy measures, such as lockdown and social distancing, have been enforced to restrict the spread of the virus (Upal, 2020). Although these measures help reduce the spread of disease, they may affect the normal economic development of the United States. According to recent data, the unemployment rate has reached 10.2% (U.S. Department of Labor, 2020). Although research has indicated that policies related to social distancing and self-quarantine are effective in reducing the spread of the virus (Lewnard & Lo, 2020), these policy measures decrease social connections, which negatively affects individuals’ psychological well-being (Whillans et al., 2017). Relatedly, about 1.4 billion children are out of school or childcare, with no access to group activities and team sports (Cluver et al., 2020). Given the closures of schools/childcare centers during the pandemic, working parents need to deal with the increased demands of homeschooling and expectations and requirements of their jobs. Parents that work in health care services or essential industries may feel even more stressed due to a lack of child care and concerns related to contamination from the work place.

COVID-19 has brought new challenges to almost every aspect of parents’ and their children’s lives. Economic stress, physical and mental health concerns, challenges in homeschooling, and balancing work and life may contribute to increased parenting stress, as well as the possibility of abuse and violence against children (Cuartas, 2020; Griffith, 2020; Humphreys et al., 2020; The Alliance for Child Protection in Humanitarian Action, 2020). Given that parental stress has increased during the COVID-19 pandemic and most children are staying home, child risk of maltreatment is predicted to be increasing (Abramson, 2020). Brooks and associates (2020) recently found that parents had more conflicts with their children and yelled at their children more often in the first 2 weeks of the pandemic than that prior to the pandemic. Newspapers reported an increase in child maltreatment during the periods of self-isolation, quarantine, and lockdowns (Cuartas, 2020). Although COVID-19 may increase risk of child maltreatment, the number of maltreatment cases may be underestimated (Merritt & Simmel, 2020), because such reporting relies mainly on child welfare workers, teachers, doctors, and other professionals, and stay-at-home orders minimize their direct interactions with children (Welch & Haskins, 2020). In addition, home visits and in-home investigations, as well as in-home services, may also be limited during the pandemic, which furthers the underestimate (SAMHSA, 2020). Such an underestimate might have occured in some states (e.g., Wisconsin, Oregon, Pennsylvania, and Illinois) that reported a 20% to 70% drop in maltreatment cases reported in March 2020 (Welch & Haskins, 2020). A reported decrease does not indicate that children at risk are safe at home, for reasons just noted. In fact, the rates of child maltreatment usually increase during pandemics or emergencies (Peterman et al., 2020; Seddighi et al., 2019). Thus, an understanding of heightened risk of child maltreatment from a theoretical perspective is needed to inform practice and to prevent child maltreatment.

This conceptual article uses family stress theory to explore risk and protective factors associated with heightened risk of child maltreatment, and to inform child welfare researchers and practitioners how to enhance child safety during the COVID-19 pandemic. This article also provides suggestions for reducing family stress and increasing parental resources, developing positive perceptions, and actively coping to mitigate risk of child maltreatment, in order to better protect children during the pandemic, as well as to inform future research and practice.

## Family stress theory

**Figure 1.**
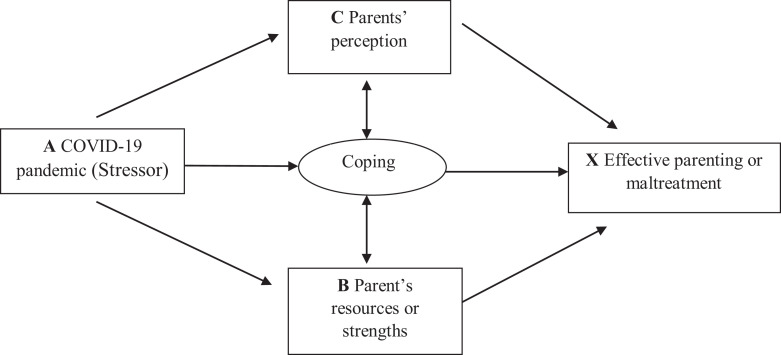
Family stress model and COVID-19 pandemic, adopted from Hill (1949).

As early as the 1930s, Angell (1936) and Cavan and Ranck (1938) used family stress theory to study how families dealt with the loss of household income and the stress associated with unemployment. Family stress theory was developed by Hill (1949) in his study of wartime separation and reunion. Hill (1949) identified a roller-coaster pattern of adjustment in reunited families, which involved initial disorganization, followed by recovery, and reorganization. This roller-coaster pattern can also be seen in the current COVID-19 situation given that many people lost their job during the pandemic in the U.S. Hill’s ABC-X model (see Figure 1) of family stress is the foundation of the current family stress theory. In this model, factor A refers to the stressor, which is the life event or occurrence causing a change in the family’s equilibrium. Factor B stands for the resources or strengths that are used to help people deal with stress. Factor C represents family members’ perceptions of the causal event (i.e., how they define the event). The X factor is the outcome of the stress or crisis, which follows the application of coping strategies.

A stressor is the first element of the ABC-X model. Hill (1949) conceptualized stressors as events or circumstances that place pressure for change on a family system. Common stress events for a family might include daily struggles with schedules, developmental transitions, unemployment, poverty, disease, divorce, and the long-term demands of parenting (McKenry & Price, 2005). This stress leads to a crisis point, and when people cannot handle this stress well with their internal coping skills or external resources, the stress then extends to become a challenge for the family. To avoid this stress contagion, parents must have adequate internal coping skills and resources to keep stress at a manageable level. Stressors will not only bring about feelings of tension for parents, but also will change the parent–child relationship and family dynamics (McKenry & Price, 2005).

The second element of family stress theory is parental resources. Once stress is experienced, the resources available to a family will determine many of the strategies used to cope with the stress or overcome difficulties. Resources are individuals, families, or larger social networks that are valuable supports to help people cope. Resources can be financial; community-based programs, such as recreation programs, parks and leisure-time activities; or other stress managing programs, such as therapy, parenting support groups, and respite care. To help reduce the number of potential stressors, external social support helps increase parents’ capacities to cope with stress and reduce depression (Koeske & Koeske, 1990). Personal resources include personal traits, characteristics, health, education, parenting experience, as well as psychological qualities, such as self-esteem and self-image, which may affect parenting styles.

The concept of a stress event is in some ways neutral, as both positive and negative events can be stressors. When it is a negative event, the stressors can be a challenge for families. As to the third element, parents can construct their own perceptions or opinions as to whether stressor events are positive or negative. Individuals’ perceptions of a given event are affected by their stage of development, early experiences, life views, and personal dispositions. When a stressful event occurs, people tend to appraise the event subjectively so that some may view the stressor in a positive framework, considering the event as a challenge or even as an opportunity to drive them to a new level of achievement. Others will view the same circumstance in a negative framework and consider the event as a crisis. In this sense, a person’s psychological responses to stressors are based on their cognitions and coping strategies (Folkman, 1999). Individual’s coping strategies are also affected by personal tendencies and characteristics, such as neuroticism, openness to new experiences, and life attitudes. Based on the interaction of stressors, a parent’s resources, and perceptions, a parent will develop their own coping strategies to deal with stressors. The most common types of coping are cognitive- or appraisal-focused, problem-focused, and emotion-focused (Folkman, 1999).

A crisis is believed to include an extreme stress response when individuals’ resources are insufficient to help to cope with stressors and they do not appropriately perceive the stressors. When parents think that they are going through a crisis, this perspective will affect all aspects of their normal life. On the emotional level, parents that feel overwhelmed by the pressure of dealing with a crisis may feel depressed. In turn, parents’ performance in the community and workplace will also be impacted by their poor mental status. On the behavioral level, when parents are experiencing a high level of stress, they may be more likely to vent their negative emotions through improper behavior, such as physical or verbal abuse (Rodriguez, 2010). Families with histories of domestic violence or child maltreatment are likely to express these negative coping tactics when under stress (Usher et al., 2020).

Because family stress theory explains working mechanisms of a family, this theory helps explain how parents’ behaviors arise during the COVID-19 pandemic. When facing challenges or crises brought by the pandemic, parents’ resources, perceptions, and coping strategies determine parenting effectiveness and parenting behaviors.

## Family stress theory and risk of child maltreatment during the COVID-19 pandemic

### Stressors associated with COVID-19

Although the impacts of this pandemic vary depending on contextual stressors that family members experience (Coyne et al., 2020), the COVID-19 pandemic has brought significant multi-layer stress for families (Phelps & Sperry, 2020). These stressors and their impacts on family lives may also vary by an individual’s demographic characteristics, such as race/ethinicity (Goosby et al., 2012), and socioeconomic status (Emmen et al., 2013). The following are examples of significant stressors that COVID-19 brings to families.

#### Physical health and mental health concerns

Due to the rapid transmission of the virus and its serious health implications, family members may be afraid of and have concerns related to COVID-19. Parents need to handle fear and anxiety in these uncertain times, and they may also need to inform and explain to children about the COVID-19 pandemic. When parents cannot handle the challenges well, they may experience psychological distress. For low-income families, parents are more likely to work as frontline workers in essential services, such as grocery stores, but they may not have the necessary safety equipment to protect themselves from possible contact with the virus (Kantamneni, 2020). In addition, prolonged social distancing and quarantine may lead to increased incidences of depression (Safai, 2020), alcohol abuse (Sganga, 2020), intimate partner violence (IPV; SAMHSA, 2020), and trauma (Crayne, 2020), which are risk factors for child maltreatment. For families that have parents or family members who have died or were ill due to COVID-19, parents may experience more psychological distress and have to deal with grief during these uncertain times.

#### Economic stress

The global COVID-19 pandemic has had profound effects on family economic well-being. In just 4 weeks after the declaration of a national emergency in the U.S., more than 30 million people lost their jobs (Rugaber, 2020). The material and financial hardships due to the pandemic are hitting low-income, Black, and Hispanic families especially hard (Jenco, 2020). Even after the peak has passed, the pandemic will continue to impact the economy and employment, and millions of people will likely have no stable income in the near future (Crayne, 2020). Losing income or lacking stable income, families will not be able to afford rent, utilities, internet, or other daily expenses during and even after the pandemic. Loss of a job often means loss of medical insurance, which will only exacerbate financial pressure, particularly for families that had a low income. Many previous studies have shown that poverty or unemployment is significantly associated with increased family stress (e.g., Howe et al., 2012; Pignault & Houssemand, 2018; Thompson et al., 2013).

#### Challenges in homeschooling

Even if parents do not face immediate physical or mental illness or unemployment, many are likely to experience an unprecedented daily stress associated with the challenges of homeschooling (Schneider et al., 2017). With the recent sharp increase in diagnosed COVID-19 cases, many schools have developed a remote learning format so that students stay at home and learn online during the pandemic. This new change brings challenges for full-time or part-time working parents. Since many schools are closed, families have responsibilities to teach and support children academically in addition to providing emotional support, which were originally charges of the schools. However, effective teaching and learning may not be fostered in families, and for children that have pre-existing behavioral and mental health issues, parents may not be able to deal with their problems adequately. Given that the pandemic has created significant socioemotional and financial stress on low-income families, it has been difficult for children in these households to focus adequately on academic tasks (Phelps & Sperry, 2020). The challenges of keeping children busy and safe at home are also exacerbated for those living in low-income and crowded households (Cluver et al., 2020). Furthermore, low-income families may have limited access to the internet, or parents may have limited time for homeschooling, and educational resources and knowledge, which may potentially increase risk of child maltreatment. Even when schools provide online learning opportunities for children, many essential workers must leave home and cannot support children for online learning.

#### Marital conflicts and intimate partner violence (IPV)

Prior research has found that increased marital dissatisfaction is associated with several factors, including financial issues (Amato & Rogers, 1997; Neppl et al., 2016), and physical (Daniel et al., 2009) and mental illness (Jarnecke et al., 2016), all of which may be possible results of the COVID-19 pandemic. Many people have reported that COVID-19 quarantines have harmed their marital relationship, which could potentially lead to a higher divorce rate once divorce courts are again fully open (Puente, 2020). For those that were experiencing IPV before the pandemic, the likelihood of exposure to domestic violence is increased due to quarantine (Fegert et al., 2020). This makes it difficult for victims to ask for help and escape from abusive partners. There has been a rise in IPV worldwide during the period of pandemic lockdown (Graham-Harrison et al., 2020). Such an increased IPV rate results in increased substance abuse (Usher et al., 2020), which increases the risk of child maltreatment (Fegert et al., 2020). In addition, exposure to IPV would also increase the likelihood of physical, sexual, and emotional violence against the children (Fegert et al., 2020).

#### Intensified parent–child relationship

The quarantine may increase tensions between parents and children because they spend more time at home interacting with each other. Without many opportunities to play with peers, children’s social needs cannot fully be met, which may intensify the relationship with their parents. Prior research shows that the effects of disasters, such as Hurricane Katrina, can worsen many pre-existing problems for children and their relationships with parents (Shapiro et al., 2006). Therefore, quarantine may increase the frequency or intensity of cycles of interpersonal violence between parents, their partners, and their children. This situation may become worse especially for children that already had poor relationships with their parents, had behavior problems, mental illness, or other special needs before the pandemic. If parents do not have effective coping strategies and parenting skills, the COVID-19 pandemic may increase children’s risk of maltreatment and adversity (Phelps & Sperry, 2020).

### Parents’ internal and external resources

#### Internal resources

An individual’s internal resources include personal traits, life experiences, economic well-being, knowledge of parenting, health, and resilience (White & Wu, 2014). All of these factors comprise parents’ internal resources that they may use when under family stress, and they contribute to the development of their parenting styles. Baumrind (1966) proposed three parenting styles: permissive, authoritarian, and authoritative. According to Baumrind (1967), a permissive parent attempts to behave in a nonpunitive, affirmative, and accepting manner towards the child’s behaviors, opinions, and desires. Authoritarian parents are those that shape, control, and appraise the attitudes and behavior of the child according to a standard of conduct, usually an absolute standard. An authoritative parent directs children’s activities in a rational manner, and they are responsive to the child’s emotional needs while having high standards. The latter is considered the most effective parenting style, because it makes demands that fit with a child’s ability to be responsible for their own behavior. Because parents have their own styles of parenting, different parents may respond with different parenting practices when facing the same challenges.

#### External resources

One of the most significant resources for parents is their social support, which has three components: Emotional support that makes people feel they are cared for and loved; esteem support so people believe they are valued; and network support consisting of a defined position in a complex of communication and mutual obligations (Cobb, 1979). In addition, financial support is essential to deal with financial challenges that are brought by stresses. For parents, formal supports include those received from their community, children’s schools and teachers, colleagues and the government, and informal supports include those provided by family members and friends. Once they face difficulties in parenting or other aspects of their lives, obtaining more resources and advice from others helps parents overcome difficulties and more effectively parent their children. On the contrary, lacking social support may increase parents’ anxiety and stress, which could negatively affect their parenting behaviors, such as increasing psychological aggression, corporal punishment, or neglectful behaviors.

Because of keeping a safe social distance, the COVID-19 pandemic may influence parents’ formal or informal social support systems regardless of the parents’ social-economic status. In addition, parents may not be able to acquire adequate support from children’s schools and teachers in terms of homeschooling suggestions, and parents may not receive adequate support from colleagues, family members, and friends. The pandemic likely affects families of low socioeconomic status most significantly (Fegert et al., 2020). In terms of formal support, the current financial compensation from the U.S. federal government is not sufficient for parents that have been laid off during the pandemic. Therefore, the current erosion of social support systems and the explosion of multiple stressors may increase vulnerabilities of parents, which may, in turn, lead to increased violence against children at home (Cicchetti et al., 2000).

### Parental perceptions

Whether the COVID-19 pandemic becomes a crisis for a family depends on how parents construct their perceptions of a stressful event. The impact of the pandemic on parental perceptions is highly heterogeneous (Vinkers et al., 2020). Miragoli et al. (2018) proposed that parents’ social-cognitive capacities are the basic building blocks underlying their parenting practices, according to the social information processing model. Parents’ social-cognitive capacities, as well as the impacts of the pandemic, are affected by their personal characteristics, living conditions, health status, income, previous life experiences, uncertainty about the future, and the level of perceived social support (Southwick & Charney, 2012). For example, if a parent has a positive life attitude and health condition, he/she may view the pandemic from a positive perspective and may not be seriously impacted by the pandemic. The parent may treat the quarantine as a good opportunity to spend more time with the child, and she/he is more likely to show a positive perception toward stressors associated with COVID-19 than parents with a negative life attitude and/or poor health. Research has shown that a positive attitude toward COVID-19 and confidence in epidemic control are associated with lower levels of depression and higher levels of happiness (Lu et al., 2020). In this case, more effective parenting practice will be used rather than child maltreatment.

Before taking any parenting action, parents usually go through a cognitive process. Even if many parents seem to take actions without consciously thinking through potential actions and consequences, they will go through a cognitive process unconsciously. First, they perceive what has happened or what is happening in the family during the pandemic. Second, they interpret the current situation they are facing, which is affected by their personal characteristics. Third, they develop certain coping strategies (e.g., cognitive or appraisal focused coping strategy) based on their social supports or resources and perceptions. When a child shows behavior problems, parents’ tolerance may be affected by their internal and external resources mentioned above, which further affect their understanding of the child’s behaviors. It is important to mention that previous studies found that people’s characteristics may be changed after some crisis (Peterson & Seligman, 2003). For example, Peterson and Seligman (2003) found that some character virtues, such as hope, kindness, love, spirituality, and gratitude, increased after the 9/11 tragedy. Therefore, a parent’s perceptions toward stressful events could be changed via certain interventions that are focused on an individual’s cognitive processes when facing stressors (Ngai et al., 2016).

### Coping strategies

Both parents’ resources and perceptions in the face of the COVID-19 pandemic affect their coping strategies. Use of positive coping strategies helps parents build distress tolerance, increase social support, make positive meanings, and take goal-directed and value-driven actions during the COVID-19 pandemic (Polizzi et al., 2020). Finding ways to engage with and appreciate life during a pandemic reduces posttraumatic stress symptoms and increases psychological well-being (Dekel et al., 2016). With positive emotions, individuals can rebound from negative experiences more easily (Fredrickson et al., 2003), and free-up cognitive resources to contend with everyday stressors and adjust to fluctuating situational demands (Bonanno et al., 2010).

When facing the stressor of COVID-19, parents may use different types of coping strategies. If they use cognitive- or appraisal-focused coping strategies, they may change their thinking about COVID-19, and re-assess the impacts of the pandemic on their family lives. If parents use a problem-focused coping strategy, they will find ways to deal with the challenges that the pandemic brought to their family. For example, they may find ways to become employed, if they lose a job during the pandemic. If parents use emotion-focused coping strategies, they may seek emotional support or counseling services when they are experiencing negative emotions. Given the complex and increased stress during the COVID-19 pandemic, parents that have few appropriate coping strategies may be more likely to maltreat a child (Abramson, 2020; Lawson et al., 2020). Previous literature has shown that abusive parents report a significantly higher level of parenting stress (Miragoli et al., 2016), and this may interfere with their ability to cope effectively with parenting difficulties, thus increasing the likelihood of child maltreatment (McPherson et al., 2009). Therefore, increased stress, limited support, as well as parents’ perceptions during the pandemic may lead to the increased use of inappropriate coping strategies while parenting, such as emotion- and avoidance-oriented strategies (Chen et al., 2016).

### Increased risk and decreased report

Although there is an increased risk for child abuse and neglect during a pandemic, child maltreatment may be underestimated given the challenges child protective services (CPS) are facing. When childcare centers or schools are closed and there is reduced surveillance (Herd et al., 2020), mandated reporters, such as teachers and doctors, have less in-person contact with children, which could lead to less reporting of child maltreatment. Quarantine with family members may increase the risk of child sexual abuse, as this is mostly perpetrated by people known to the victim (Finkelhor, 2009). By the end of March of 2020, there was a 22% increase in monthly calls to the National Sexual Assault Hotline, and 67% of children that reported identified their perpetrator as a family member (Kamenetz, 2020). In the meantime, CPS are also facing additional challenges. When receiving a child maltreatment report, social workers investigate and make decisions as to whether the case is substantiated. However, due to the coronavirus, social workers may not be able to conduct a thorough investigation through home visits and talking to parents and child, so there are lowered chances to determine whether maltreatment has occurred (Herd et al., 2020).

## Implications for practice

Family stress theory provides a theoretical perspective to understand the impacts of the COVID-19 pandemic on increased risk of child maltreatment. To better address challenges related to COVID-19 in family settings and protect children during the pandemic, we must understand the nature of the stressors, make greater efforts to provide continuous resources and supports, build parents’ positive perceptions towards stressors and other events during COVID-19, and encourage them to employ appropriate coping strategies. In this way, effective parenting practices can be developed to decrease the risk of child maltreatment. We recommend that practitioners that serve children and families, and parents that have children at home, consider the following suggestions. To begin, as the family stress theory treats the family as a holistic unit, we recommend using a family-centered perspective instead of parent-centered or child-centered perspectives to address family stress, improve parents’ capability, and reduce risk of child maltreatment (Xu et al., 2020).

A major stressor revolves around physical health and mental health concerns. Parents and children should be educated to use effective measures to protect themselves and their children from COVID-19. For example, parents should actively learn and use strategies of self-protection available online regarding staying healthy during COVID-19, such as wearing masks, keeping social distances, and encouraging their children to do so, as well. To reduce children’s anxiety and fear, parents should educate children appropriately on how to protect themselves by providing knowledge of COVID-19, and sharing emotions related to COVID-19 with their children. To address parents’ and children’s mental health concerns, we recommend offering more emotional supports via multiple sources, such as telemental health services, and peer support groups, to parents and children in need. Both federal and state legislation and regulations have changed in response to the pandemic to increase availability of telehealth/telemental health services (American Psychiatric Association, 2020). In many states, health care providers have used the telehealth/telemental health services via FaceTime, skype, or other apps, which increase availability of health care services during the pandemic (Conrad et al., 2020). Meanwhile, parents and children should be encouraged to maintain and strengthen social relationships with significant others, such as relatives, friends, and colleagues, to build social support while keeping safe social distance. Extra services that can build social supports or access to social services should be provided to vulnerable families that have been struggling with pre-existing challenges, such as poverty, social isolation, IPV, substance abuse, and child maltreatment prior to the pandemic.

The importance of having emergency funds and a stable financial status has been highlighted during the COVID-19 pandemic (Sherraden et al., 2020). In terms of family economic stress, the U.S. federal government has provided financial supports to individuals, such as a $1,200 stimulus check, and offering compensation and medical benefits for federal employees. However, more attention should be paid to low-income parents that desperately need more financial support and emergency funds to survive this pandemic (Sherraden et al., 2010).

In terms of the challenges of homeschooling, more opportunities to work from home and more flexible work schedules should be provided to accommodate parents’ needs. Parent support groups could be developed, in which families pair with others to teach children together. In the upcoming academic year, many children and families may choose the online learning mode. Given that many parents need to work on-site and full time, the digital learning environment may create extra challenges for parents since it may require parents to help young children properly set up for such learning. Thus, social workers or child welfare professionals should provide more child care/homeschooling support to parents by collecting information online regarding tips for providing child care or homeschooling. Providing ideas about activities and resources to support learning at home will help to decrease parenting stress during the pandemic.

To improve marital relationships between partners in a two-parent household, parents should give each other some separate time, if desired, and recognize and appreciate positive life changes. To improve the relationship between the child and parent(s), parent(s) are encouraged to remain calm, spend individual time with the child, and praise the child when they behave well (United Nations Children’s Fund, 2020). Families should be more innovative in seeking supports to have better access to social services and couple’s therapy. For example, parents could use online consulting services for support when coping with partner or child related issues. It is also important to advocate that free services should be provided to low-income parents in this special time.

To reshape parents’ perceptions towards COVID-19 related stressors, it is important for parents and children to make meaningful interpretations of COVID-19, and identify their family strengths and resilience during this pandemic. When providing parenting interventions to parents, the interventions could focus on cognitive-behavior changes to help parents re-think about COVID-19 or their life experiences from a new and positive perspective, so that they can explore possible beneficial impacts of these life events on their psychological well-being. Effective coping strategies should also be provided through parenting interventions for parents to learn positive ways to cope with stress. More importantly, to address parents’ negative perceptions, more concrete support and resources should be provided for them, as their perceptions toward stress might be changed when support is increased (Pinto et al., 2019).

To foster effective and positive coping strategies, more skills-based interventions should be implemented for parents. By participating in such interventions, parents can learning different coping strategies, such as cognitive- or appraisal-focused, problem-focused, and emotion-focused strategies, so that they can use these skills to deal with family stress during the pandemic. Furthermore, preventive parenting interventions should be implemented to target high risk families so that ineffective parenting practices may be reduced. Interventions could be offered for free online during this pandemic to ensure child safety.

By understanding the nature of the stressors, increasing parental resources, and changing parental perceptions and coping strategies, we may reduce the risk of child maltreatment (Abramson, 2020; Lawson et al., 2020; Ngai et al., 2016). As COVID-19 has transformed our lives in several ways, and it may become a new normal for us, parents and children need to adjust their perceptions about life changes caused by COVID-19 and make corresponding adaptations. Although services should be provided to all families regardless of socioeconomic status, more services should be available to high risk and low-income families. Practitioners that serve this population may adapt social services using new technologies (e.g., virtual meetings) and new approaches (e.g., online services). But while practitioners are making such adjustments, it is important that they consider whether these services are accessible to parents and children in need.

To reduce risk of child maltreatment during and after a pandemic, a public health prevention approach (National Center for Injury Prevention and Control, n.d.) would be beneficial. The first step is to recognize increased risk of child maltreatment during the pandemic. The second step would be to identify risk and protective factors associated with child maltreatment during the pandemic. Our theoretical discussion, based on the family stress theory, has pointed out potential risk and protective factors, which could serve as a framework for practitioners. The third step would be to develop and test prevention strategies. In addition to addressing the concern of the underestimation of the incidence of child maltreatment, more ongoing training should be provided to mandated reporters, such as teachers and health care professionals, to identify risk factors of child maltreatment when seeing children virtually. Meanwhile, since fewer opportunities of direct interactions between children and mandatory reporters exist during the pandemic, the function of non-mandated reporters of child maltreatment becomes more important. Social support between neighbors should be strengthened, as such, neighborhood collective cohesion is beneficial in terms of protecting children at a community level. As COVID-19 does not appear to be ending soon, our recommendations can be implemented by individual families first, then by more broadly community led effort. A rapid prevention strategy based on these practices could be published and used as a reference in the face of COVID-19, or other future disasters.

## Implications for research

Our theoretical discussion based on the family stress theory also points to directions for future research to understand risk of child maltreatment during a pandemic or other types of long-term emergencies. Future observational research should collect cross-sectional and longitudinal data to examine relationships among identified COVID-19 related stressors, parental resources, parental perceptions, coping strategies, and risk of child maltreatment. Observational studies can examine (1) how COVID-19 related stressors are associated with risk of child maltreatment, (2) how this relationship is indirectly affected by other factors, such as parental coping strategies, and (3) whether parental resources and parental perceptions moderate the relationships between COVID-19 related stressors, coping strategies, and risk of child maltreatment. When examining these relationships, special attention should be paid to cultural differences in parenting practices and racial/ethnic disparities. Disparities in access to social services and children’s outcomes should also be examined by race/ethnicity, socioeconomic status, and immigration status. In addition, future studies could compare different effects of various COVID-19 related stressors, parental resources, parental perceptions, and coping strategies on different types of child maltreatment, such as physical abuse, sexual abuse, neglect, emotional abuse, because the etiology of these subtypes of child maltreatment is different.

More importantly, intervention research should be conducted to reduce COVID-19 related stressors and their effects on family and child well-being during and after this pandemic. This theoretical framework points to means of intervention, such as reducing COVID-19 related family stressors, improving parental resources, changing parental perceptions, and developing positive coping strategies. In addition to these intervention components, relying on technologies, such as computer or phone apps, is an innovative approach to delivering interventions. Researchers should examine whether these types of interventions are effective in decreasing parental stress and reducing risk of child maltreatment. Also, researchers could examine whether delivering interventions via computer or phone apps is as effective as delivering in person. Because families of differing race/ethnicity and socioeconomic status may face different challenges, future studies can also examine and compare the effectiveness of these interventions across these groups. In addition, more culturally sensitive and tailored interventions should be developed for families with children that have experienced maltreatment prior to the pandemic, children with disabilities, children of immigrants, and children of color.

The COVID-19 pandemic has not only transformed our lives, but also has exacerbated inequality and disparities across race/ethnicity, income, gender, and immigration status. Intersectionality refers to complex, irreducible varied variables, such as race/ethnicity, gender, class, and their effects on individuals in a specific context (Nash, 2008). Different from the typical family that we discussed above, families under other conditions have unique experiences in their daily life, health, and education. Future researchers should not use just intersectional perspectives to examine critical differences in data drawn from observational research and in the effectiveness of interventions, but also to explore the diversity of families and their unique stressors and coping strategies.

## Conclusion

This article uses the family stress theory to illustrate how the COVID-19 pandemic has potentially increased family stress, and how parental resources, perceptions, and coping strategies might interactively contribute to ineffective parenting practices and increased risk of child maltreatment. This article also shows that a similar conceptual framework might be used to reduce risk of child maltreatment, as well as to identify directions for future research. In addition, practitioners may explore and develop more effective interventions based on the implications of this study to better help parents and families deal with the challenges brought by the COVID-19 pandemic.
